# Emotional intelligence buffers the effect of physiological arousal on dishonesty

**DOI:** 10.3758/s13423-017-1285-9

**Published:** 2017-04-13

**Authors:** Andrea Pittarello, Beatrice Conte, Marta Caserotti, Sara Scrimin, Enrico Rubaltelli

**Affiliations:** 1 Department of Psychology, Faculty of Behavioral and Social Sciences, University of Groningen, 9712 TS Groningen, The Netherlands; 2 Department of Psychology and Swiss Center for Affective Sciences, University of Geneva, Geneva, Switzerland; 3 Department of Developmental and Socialization Psychology, University of Padova, Padova, Italy

**Keywords:** Skin conductance, Unethical behavior, Emotional intelligence, Arousal, Cheating

## Abstract

We studied the emotional processes that allow people to balance two competing desires: benefitting from dishonesty and keeping a positive self-image. We recorded physiological arousal (skin conductance and heart rate) during a computer card game in which participants could cheat and fail to report a certain card when presented on the screen to avoid losing their money. We found that higher skin conductance corresponded to lower cheating rates. Importantly, emotional intelligence regulated this effect; participants with high emotional intelligence were less affected by their physiological reactions than those with low emotional intelligence. As a result, they were more likely to profit from dishonesty. However, no interaction emerged between heart rate and emotional intelligence. We suggest that the ability to manage and control emotions can allow people to overcome the tension between doing right or wrong and license them to bend the rules.

People encounter ethical dilemmas almost every day. They can receive extra change at the grocery store, or they are tempted to steal office supplies at work. In these situations, they face two opposite desires: serving their self-interest, and maintaining a positive self-concept. Accordingly, they cheat to the extent to which they can balance these opposite motivations (Ayal & Gino, 2011; Barkan, Ayal, Gino, & Ariely, 2012; Mazar, Amir, & Ariely, 2008).

Recent work examined the cognitive and contextual factors that enable people to reduce this tension and bend ethical rules, such as self-serving justifications and ambiguous rules of conduct (Ayal, Gino, Barkan, & Ariely, 2015; Pittarello, Leib, Gordon-Hecker, & Shalvi, 2015; Schweitzer & Hsee, 2002; Shalvi, Dana, Handgraaf, & De Dreu, 2011; Shalvi, Gino, Barkan, & Ayal, 2015). However, the emotional mechanisms underlying tension reduction have been largely ignored.

We aim to fill this gap and propose that the way people manage and control emotions (i.e., emotional intelligence) will allow them to overcome the discomfort that arises from the desire to do wrong while at the same time maintaining a positive self-image. Specifically, we suggest that people who can successfully manage and control their emotions will be less affected by the arousal elicited by ethical dilemmas (Gu, Zhong, & Page-Gould, 2013; Wilkinson, 1987) than those who are less capable to do so. As a result, they will be more likely to serve their self-interest dishonestly.

## Physiological costs of lying

Moral concerns can be a source of distress, because they confront people with desires that are inconsistent with their moral principles (Allport, 1955; Hochman, Glöckner, Fiedler, & Ayal, 2015; Rosenberg, 1979; Wilkinson, 1987). These concerns trigger a stress reaction linked to the autonomic nervous system (ANS) response. This reaction can be registered in the form of physiological arousal in different somatic systems. Two well-established measures of arousal are the simultaneous registration of changes in heart rate and electrodermal activity.

In normal adults, heart rate (HR) depends on the pacemaker activity of the sinoatrial node cells and constantly varies in response to a number of factors, including cognitive effort and emotional stimulation (Kreibig & Gendolla, 2014). The sinoatrial node cells are innervated by the sympathetic nervous efferents and by the parasympathetic fibers of the vagus nerve, which exert a speeding-up and a slowing-down influence on the heart, respectively. Increased sympathetic activity, which occurs during a challenge or a psychological stress (Brosschot & Thayer, 2003), correlates with acceleration in heart rate. Such acceleration reflects higher arousal (Appelhans & Luecken, 2006).

Likewise, changes in electrodermal activity in response to a stressor represent a reliable index of sympathetic activation (Boucsein, 1992; Kreibig & Gendolla, 2014). Skin conductance level (SCL) is an electrodermal measure caused by the activity of sweat glands that are innervated solely by the sympathetic branch of the autonomic nervous system (SNS). Because SNS activity is predominant when responding to emotional challenging stimuli, changes in SCL are a particularly useful indicator of emotional arousal (Boucsein, 1992).

Hence, both somatic systems respond to stress with a substantial increase in their activity and share some common representation in the brain. Despite these similarities, the brain patterns that predict these two autonomic responses are largely distinct (Eisenbarth, Chang, & Wager, 2016). These observed differences could reflect different involvement of parasympathetic and sympathetic responses (Berntson et al., 1997). Whereas an increase in SCL is related exclusively to a sympathetic response, HR also is subjected to a more rapid parasympathetic contribution (Berntson et al., 1994; Eisenbarth, et al. 2016). For these reasons, it is important to measure multiple types of autonomic activity to clarify the role of different systems and how they play out when it comes to stressful decisions.

Recent work suggests that stress and arousal are “instrumental in driving ethical behavior” (Teper, Zhong, & Inzlicht, 2015, p. 2). Accordingly, Teper and colleagues (2011) found that participants’ levels of arousal corresponded to lower rates of unethical behavior, and Gu and colleagues (2013) showed that participants who listened to faster (vs. slower) heart beats were less likely to act dishonestly. The idea behind these findings is based on the stress and coping theory (Lazarus & Folkman, 1984), which suggests that, in stressful situations, people attempt to remove the source of stress or regulate stressful emotions via coping strategies. When it comes to ethical dilemmas, resolving such stress can either lead people to do the right thing or lie (Gu et al., 2013). Finally, Dienstbier and Hunter (1971) found that people cheated more when they could attribute stress symptoms to external causes. If arousal curbs dishonesty, it is reasonable to expect its effect to be weaker among people who are generally less impacted by it, such as those who can successfully cope with aversive states and are more capable to manage their emotions.

## Emotional intelligence and coping strategies

Although everybody experiences emotions, people differ in the extent to which they identify, manage, and control them. One construct widely used to account for these differences is trait emotional intelligence (henceforth: trait EI). According to Petrides, Pita, and Kokkinaki (2007), trait EI represents a set of emotional perceptions located at the lowest level of the personality hierarchies. This definition of trait EI recognizes the subjective nature of human emotional experience and is concerned with people’s perceptions and management of their own and others’ emotions.

It has been shown that people differ in how they process, use, and manage affective information both of interpersonal and intrapersonal nature (Petrides & Furnham, 2003). For instance, people with high trait EI are better at coping with stressful situations and use more adequate emotion regulation strategies than people with low trait EI (Mikolajczak, Nelis, Hansenne, & Quoidbach, 2008; Petrides, Sangareau, Furnham, & Frederickson, 2006). Additionally, trait EI is related to different physiological measures. For example, individuals with high (vs. low) trait EI exhibited larger pupil dilation when looking at charts reporting the performance of stock funds (and were more likely to invest even when the fund had recently lost value; Rubaltelli, Agnoli, & Franchin, 2016). Furthermore, individuals with high (vs. low) trait EI tend to have lower levels of low frequency/high frequency heart rate ratio (an index of stress; Laborde, Brüll, Weber, & Anders, 2011) and lower cortisol response (Mikolajczak, Roy, Luminet, Fillée, & de Timary, 2007).

Based on these findings, we tested the hypothesis that greater physiological activation induced by ethical dilemmas would decrease dishonesty. However, this effect should be moderated by trait EI, in a way that individuals with high (vs. low) trait EI should be less impacted by their physiological reactions when facing ethical situations. This would allow them to distance themselves from their bodily reactions (Damasio, 1994) and license them to cheat.

## Method

### Participants

Sixty-seven university students (73% female, *M*_*age*_ = 22.37, *SD*_*age*_ = 4.98) were recruited via advertisements on campus and participated in an experimental session of ~20 minutes. The experiment was performed in individual sessions, and participants were left alone in the room during the task to reduce feelings of being monitored.

### Stimuli, apparatus, and procedure

Upon arrival to the laboratory, participants read and signed a consent form indicating that they would be partaking in a decision-making task and that their physiological measures would be recorded during the session. HR and SCL were measured in a standardized fashion using a multimodality, physiological monitoring device that encodes biological signals in real-time (ProComp Infiniti, Thought Technology; Montreal, Canada). To obtain HR, we recorded photoplethysmography—a simple and noninvasive technique used to detect blood volume pulse (BVP). Photopletysmograph sensor was attached to the thumb fingertip of the participant’s nondominant hand, and BVP signal was processed via a 12-bit analog-to-digital converter with a sampling rate of 256 times per second and stored sequentially for analysis. HR data recorded during each phase were computed and artifacts were controlled for (see *Supplementary Analyses* for additional details).

SCL was recorded using two Ag/Ag-Cl gel-less electrodes attached to the medial phalange of the second and fourth fingers of the participant’s nondominant hand. Sampling for electrodermal activity (EDA) was set at 256 Hz. Skin conductance in micro-Siemens (μS) was collected at a sampling rate of 32 times per second. Separate mean HR and SCL scores were calculated for the 5-minute baseline period and the entire duration of the decision-making task.

**Fig. 1:**
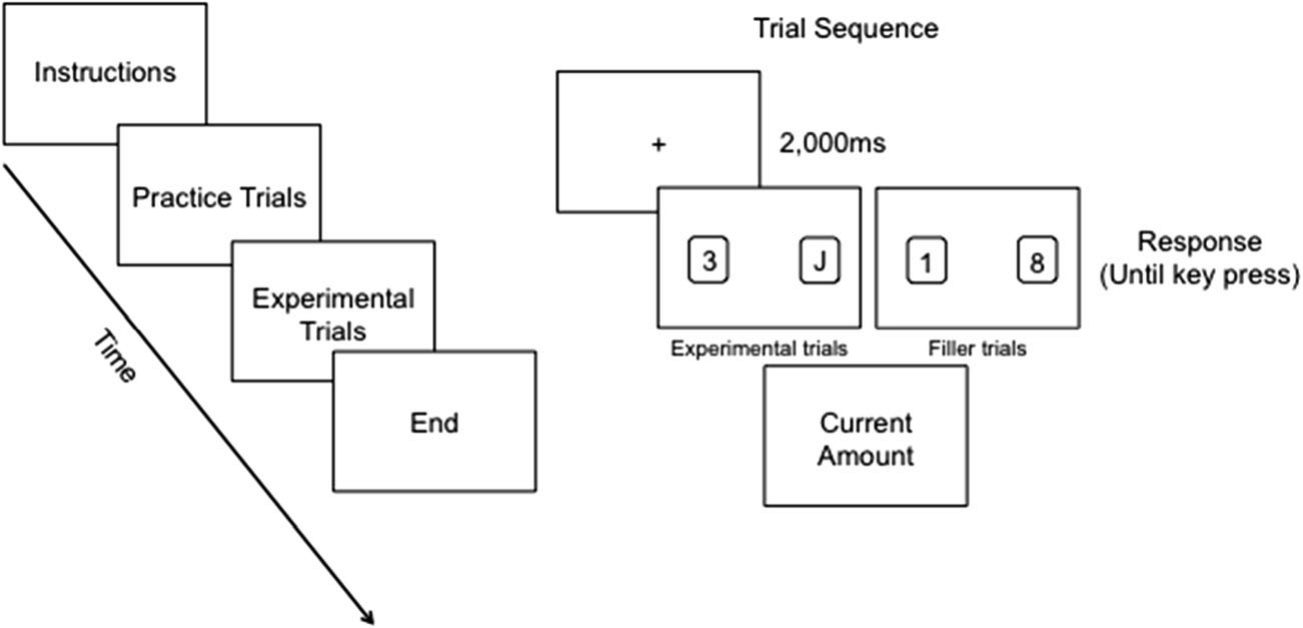
Schematic representation of the experimental procedure

Participants received an initial endowment of 60€ (~$65) and learned that at the end of the task two participants would be selected at random and win an amount of money (up to 60€) based on their decisions in the task. The task included 120 trials, preceded by a practice phase. In each trial, a black fixation cross appeared in the center of the screen for 2,000 ms, followed by two cards: one on the right side and the other on the left side of the screen. To limit the effect of time constraint (Shalvi, Eldar, & Bereby-Meyer, 2012), the cards were presented for an unlimited amount of time. In the 60 experimental trials, one of the two cards was a number (1-10), whereas the other was a “J” (Joker). In the 60 remaining filler trials, both cards were numbers. Participants were instructed to press the “m” button on the keyboard every time one of the two cards was a “J” and the “b” button every time both cards were numbers. Participants further learned that they would lose 1€ whenever they pressed the “m” button and that they would lose no money whenever they pressed the “b” button. After clicking either “m” or “b,” participants saw their current payoff amount (for a similar procedure see Motro, Ordóñez, Pittarello, & Welsh, 2016; Pittarello, Motro, Rubaltelli, & Pluchino, 2016). This setting presented an ethical dilemma: participants could be honest, report correctly that a Joker appeared on the screen, but lose 1€. Alternatively, they could cheat and fail to report a Joker when it was actually presented and lose no money (Fig. 1). For robustness purposes, the location of the Joker was counterbalanced within the trials. Including filler trials allowed us to rule out the possibility that cheating resulted from simple mistakes or inattention.

Because there were 60 experimental trials, an honest participant would end the task with 0€ (60€ - 1€ × 60 Jokers). Conversely, participants wishing to maximize their earnings could lie and fail to report a Joker when this appeared on the screen. At the end of the task, participants completed the Trait Emotional Intelligence Questionnaire Short Form (Petrides, 2009). This 30-item scale asked participants to self-report the tendency to regulate, express, and perceive their emotions. Adequate internal consistencies and broad coverage of the sampling domain of the construct have been reported (Petrides et al., 2007). Answers were provided on a 7-point scale that ranged from 1 (completely disagree) to 7 (completely agree). The measure showed a good reliability (*α* = 0.88). Upon completion of the study, participants were thanked and debriefed.

## Results

### Cheating behavior

We measured how many times participants failed to report the Joker card across the 60 experimental trials (4,020 observations). Results showed that participants cheated in 17.30% of the trials. To ensure that cheating reflected participants’ motivation to serve their self-interest rather than simple mistakes or inattention, we analyzed how many times they incorrectly reported a “Joker” card (and subsequently lost money) in the filler trials, where no Joker was displayed (i.e., “self-hurting” mistakes). In line with previous work (Motro et al., 2016; Pittarello, et al., 2016), participants were accurate in 99.3% of the filler trials, showing that errors in reporting a card are associated with the presence of a Joker; therefore, they represent actual cheating behavior.

### HR, trait EI, and cheating behavior

Two participants were excluded from the analyses due to technical difficulties in recording the HR during the task, and five participants did not complete the trait EI questionnaire. This left us with 60 participants (3,600 observations).

We conducted a repeated-measure logistic regression using R and the lme4 package (Bates et al., 2015), predicting participants’ likelihood to cheat as a function of baseline HR, gender (given our unbalanced sample), trial order (to account for the effect of time), reaction times (because previous work found that they are associated with different arousal levels, see Bradley, Greenwald, Petry, & Lang, 1992), participants’ HR during the task, trait EI (centered), and the interaction between HR during the task and trait EI. We further included the random effects of subjects, trial order, and their interaction. Results showed a significant effect of gender, *χ* ^*2*^ (1) = 68.44, *b =* −36.44, *SE =* 4.40, *p* < 0.0001, 95% confidence interval [CI] (−45.075, −27.808), showing that men were less likely to cheat than women. None of the other effects was significant, *χ* ^*2*^ (1) < 1, *ps* > 0.56.

### SCL, trait EI, and cheating behavior

**Fig. 2:**
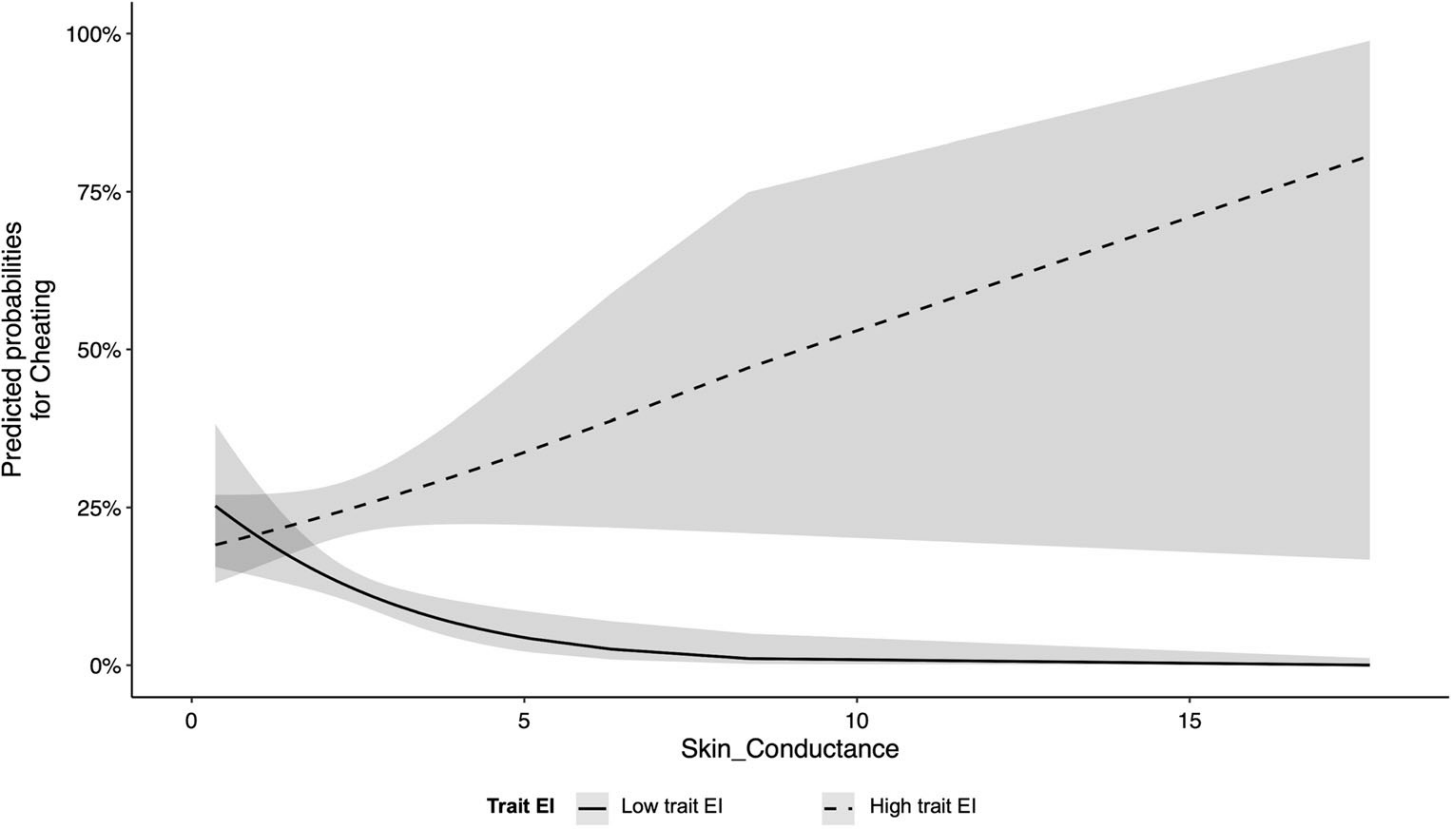
Interaction between trait EI and SCL. Trait EI was median split for ease of representation

Five participants who did not complete the trait EI questionnaire were removed from the analyses, leaving a sample of 62 participants (3,720 observations). We conducted a repeated-measure logistic regression predicting cheating behavior as a function of baseline SCL, gender, trial order, reaction times, SCL during the task, trait EI (centered), and the interaction between SCL during the task and trait EI. The random effects were subjects, trial order, and their interaction. The effect of gender was significant, *χ* ^*2*^ (1) = 17.39, *b =* −0.45, *SE* = 0.11, *p* < 0.0001, 95% CI (−0.663, −0.239), which showed that men cheated less than women. The effect of trial order was significant, *χ* ^*2*^ (1) = 6.99, *b* = 0.003, *SE* = 0.001, *p* = 0.008, 95% CI [0.001, 0.006], and indicated that cheating increased over time. The effect of SCL during the task also was significant, *χ* ^*2*^ (1) = 9.86, *b* = −0.99, *SE* = 0.314, *p* = 0.002, 95% CI (−1.600, −0.370), and showed that greater SCL during the task corresponded to a lower likelihood to cheat. Finally, the interaction between SCL during the task and trait EI was significant, *χ* ^*2*^ (1) = 8.87, *b* = 0.85, *SE* = 0.28, *p* = 0.003, 95% CI (0.289, 1.401), and indicated that as SCL during the task increased, the likelihood to cheat increased for higher trait EI (Fig. 2).

## Discussion

In ethical situations, the ability to manage emotions allows people to reduce the tension between the desire to do right and the motivation to serve their self-interest. Using the Joker task (Motro et al., 2016; Pittarello et al., 2016), we asked participants to report whether one of two cards presented on the screen was a Joker or not. Because reporting a Joker meant losing money, participants could cheat and fail to report it. We recorded HR and SCL—two measures of emotional arousal (Appelhans & Luecken, 2006; Boucsein, 1992)—and found that higher SCL corresponded to a lower likelihood to cheat. Importantly, this association was moderated by emotional intelligence: as SCL increased, participants with high emotional intelligence were more likely to cheat than those with low emotional intelligence. The finding suggests that high emotional intelligence allows people to distance themselves from their bodily reactions (Damasio, 1994; Slovic, Finucane, Peters, & MacGregor, 2007) and licenses them to do wrong. When experiencing the increase in emotional arousal, caused by a task that places the individual under stress and hence activates a defensive response, participants with low emotional intelligence were less capable of managing their emotions and preferred to reduce their cheating behavior to cope with the situation and restore homeostasis.

However, we did not find the same pattern when looking at heart rate. One possible explanation is that arousal is more closely associated with increases in skin conductance level than cardiac acceleration (Barry & Sokolov, 1993), which is a more complex measure that depends on the SNS, but it also is regulated, in part, by the PNS (Cacioppo & Tassinary, 1990).

While the involvement of both autonomic branches may cause a lower perception of emotional arousal, the activation of the single SNS, an index of SCL, induces a more intense perception of aroused bodily responses. This idea is related to the somatic marker hypothesis (Bechara, Damasio, Damasio, & Anderson, 1994), which highlights the importance of signals from the body when decisions are taken to obtain a punishment or a reward. These somatic markers have been linked to the activity of the ventromedial prefrontal cortex (VMPFC; Wright, & Rakow, 2017)—the most predictive brain pattern involved in SCL (Eisenbarth et al., 2016). Accordingly, people with high trait EI have been found to have more intense somatic activation that, as a result, induce them to regulate their emotions more effectively (Rubaltelli et al., 2016).

As mentioned earlier, the literature shows a high amount of unshared variance across HR and SCL (e.g., 86%; see Croft et al., 2004), which can be explained by largely distinct brain patterns activated by these two autonomic responses (Eisenbarth et al., 2016). SCL is linked to VMPFC, whereas HR is mostly related to the dorsal anterior cingulate cortex, which does not seem to play a key role in linking somatic responses with EI.

We believe that our results provide novel contributions to the field of behavioral ethics and emotional intelligence. Our work fits the call for a better understanding of how emotions and emotional processes affect dishonesty (Treviño, den Nieuwenboer, & Kish-Gephart, 2014). Additionally, we build on emotional intelligence literature to further our knowledge of what leads people to solve the tension that arises when the self-interest is pitted against honesty. We also extend the current literature on emotional intelligence. Conventional wisdom considers emotional intelligence as a positive trait, because it is generally associated with happiness, well-being, effective interpersonal relationships, and successful job performance (Cote & Miners, 2006; Furnham & Petrides, 2003). On the other hand, the downsides of emotional intelligence, so far, received little attention (for an exception see Austin, Farrelly, Black, & Moore, 2007). We cautiously suggest that emotional intelligence can backfire when people have the opportunity to profit from dishonesty, allowing people to rationalize on their misbehavior and frame it in a more acceptable way (Tenbrunsel & Messick, 2004). While we believe that this is an interesting perspective with real-world implications, additional research is needed to strengthen this claim.

From a theoretical point of view, it would be worth manipulating emotion management strategies to test causality and to gain insights on whether individuals can learn to downplay their emotional reactions in ethical situations. One way to do so would be to instruct participants to distance themselves from their emotional reactions and ask them to think in a very analytical and objective manner before giving them the possibility to cheat.

Because we assessed the average levels of heart rate and skin conductance during a series of ethical decisions, we cannot infer causal relationship between arousal and dishonesty. In line with Hochman and colleagues (2015), we suggest that arousal reflects the tension between doing right or wrong. It should be noted that previous work by Coricelli, Joffily, Montmarquette, & Villeval (2010) maintained that higher arousal corresponds to greater cheating. However, Coricelli and colleagues’ clever design assessed tax evasion behavior—a situation in which participants potentially could be punished, and whose identity as evaders could be made public. Future work is needed to clarify the association among emotions, arousal, and cheating. One way to do this would be to devise one-shot experiments in which arousal is measured before and after the decision to cheat, and emotional intelligence is assessed. This setting also would shed light on whether emotional intelligence acts as a pre- or post-violation strategy to cope with arousal.

In our task, participants could cheat to avoid losing money. In these situations, people are generally more likely to behave unethically compared with when the cheating would allow them to earn an equal-sized gain (Cameron & Miller, 2009; Kern & Chug, 2009; Grolleau, Kocher, & Sutan, 2016; Schindler & Pfattheicher, 2017; Shalvi, 2012). It would be interesting for future research to examine whether the buffering effect of emotional intelligence on arousal is amplified when people can bend ethical rules to increase (vs. avoid losing) their monetary endowment. Relatedly, a promising line of work would be to manipulate the ambiguity of a moral situation and measure individual differences in morality traits. A reasonable prediction would be that emotion regulation strategies would license people to cheat only when the cheating is hard versus easy to justify and would have little effect among participants with higher moral disengagement or psychopathy traits, who generally tend to suffer emotional deficits.

## Conclusions

Unethical behavior poses great threats to individuals and societies at large. It is therefore crucial to understand what leads people to act dishonestly. We suggest that emotional intelligence can license people to do wrong by reducing the tension that arises from acting upon their moral beliefs and serving their self-interest. Our results have important applied implications. For instance, managers should be aware of potential side effects of emotional intelligence when assigning ethical tasks to their employees. By doing so, they would be able to curb malpractices and promote a more ethical climate.
